# Ultra-strong diffusion-weighted MRI reveals cerebellar grey matter abnormalities in movement disorders

**DOI:** 10.1016/j.nicl.2023.103419

**Published:** 2023-04-28

**Authors:** Chantal M.W. Tax, Sila Genc, Claire L MacIver, Markus Nilsson, Mark Wardle, Filip Szczepankiewicz, Derek K. Jones, Kathryn J. Peall

**Affiliations:** a Cardiff University Brain Research Imaging Centre (CUBRIC), School of Physics and Astronomy, Cardiff University, Cardiff, UK; b Cardiff University Brain Research Imaging Centre (CUBRIC), School of Psychology, Cardiff University, Cardiff, UK; c Diagnostic Radiology, Clinical Sciences Lund, Lund University, Lund, Sweden; d Cardiff and Vale University Health Board, University Hospital of Wales Cardiff, Heath Park, Cardiff, UK; e Medical Radiation Physics, Clinical Sciences Lund, Lund University, Lund, Sweden; f Neuroscience and Mental Health Research Institute, Division of Psychological Medicine and Clinical Neurosciences, Cardiff University, Cardiff, UK; g Neuroscience Advanced Clinical Imaging Service (NACIS), Department of Neurosurgery, The Royal Children's Hospital, Parkville, Victoria, Australia; h University Medical Center Utrecht, Utrecht, The Netherlands

**Keywords:** Movement Disorders, MRI, Cerebellum, Diffusion, Microstructure

## Abstract

Structural brain MRI has proven invaluable in understanding movement disorder pathophysiology. However, most work has focused on grey/white matter volumetric (macrostructural) and white matter microstructural effects, limiting understanding of frequently implicated grey matter microstructural differences. Using ultra-strong spherical tensor encoding diffusion-weighted MRI, a persistent MRI signal was seen in healthy cerebellar grey matter even at high diffusion-weightings (b ​≥ 10,000 s/mm^2^). Quantifying the proportion of this signal (denoted $f_{s}$), previously ascertained to originate from inside small spherical spaces, provides a potential proxy for cell body density. In this work, this approach was applied for the first time to a clinical cohort, including patients with diagnosed movement disorders in which the cerebellum has been implicated in symptom pathophysiology. Five control participants (control group 1, median age 24.5 years (20–39 years), imaged at two timepoints, demonstrated consistency in measurement of all three measures - MD (Mean Diffusivity) $f_{s}$, and $D_{s}$ (dot diffusivity)- with intraclass correlation coefficients (ICC) of 0.98, 0.86 and 0.76, respectively. Comparison with an older control group (control group 2 (n = 5), median age 51 years (43–58 years)) found no significant differences, neither with morphometric nor microstructural (MD (p = 0.36), $f_{s}$ (p = 0.17) and $D_{s}$ (p = 0.22)) measures. The movement disorder cohort (Parkinson’s Disease, n = 5, dystonia, n = 5. Spinocerebellar Ataxia 6, n = 5) when compared to the age-matched control cohort (Control Group 2) identified significantly lower MD (p < 0.0001 and p < 0.0001) and higher $f_{s}$ values (p < 0.0001 and p < 0.0001) in SCA6 and dystonia cohorts respectively. Lobar division of the cerebellum found these same differences in the superior and inferior posterior lobes, while no differences were seen in either the anterior lobes or with $D_{s}$ measurements. In contrast to more conventional measures from diffusion tensor imaging, this framework provides enhanced specificity to differences in restricted spherical spaces in grey matter (including small cells) by eliminating signals from cerebrospinal fluid and axons. In the context of human and animal histopathology studies, these findings potentially implicate the cerebellar Purkinje and granule cells as contributors to the observed signal differences, with both cell types having been implicated in several neurological disorders through both postmortem and animal model studies. This novel microstructural imaging approach shows promise for improving movement disorder diagnosis, prognosis, and treatment.

## Highlights

• Ultra-strong isotropic diffusion-weighted MRI reveals signal in healthy cerebellar grey matter.
• This study applies the approach to patients with diagnosed movement disorders.
• Significantly lower mean diffusivity and higher restricted signal fraction in SCA6 and dystonia.
• This demonstrates the potential for enhancing sensitivity and specificity to small spherical spaces.
• Promising measure of cerebellar microstructural differences in movement disorders.

## 1 Introduction

Conventional MRI of brain structure is invaluable in determining macroscopic regional differences in brain volume, facilitating understanding of the pathological changes underlying a diverse range of movement disorders. (Krismer et al., 2019, Arruda et al., 2020) However, large-scale morphometric measurements provide no information of changes in the brain’s underlying microstructure, at which level multiple cell properties, packing configurations and extracellular architecture can drive volumetric differences. The most commonly applied technique for providing a more detailed description of tissue microstructure *in vivo* is diffusion MRI (dMRI), allowing for structures to be probed at much smaller scales than the imaging resolution by sensitising the signal to the random motion of water.

Investigation of brain microstructure in individuals diagnosed with movement disorders has focused predominantly on dMRI imaging changes in white matter (WM), relying on diffusion tensor magnetric resonance imaging (DTI), a dMRI technique using low-to-moderate diffusion weightings (b ​≤ 1500 s/mm^2^) whose metrics, such as fractional anisotropy (FA) and mean diffusivity (MD), provide only limited understanding of the underlying tissue properties, (Jones et al., 2013) making definitive attribution of imaging to specific microstructural compartments challenging. (Oestreich et al., 2019) However, post-mortem studies of distinct movement disorders suggest that changes in grey matter (GM) cellular properties are also of pathophysiological importance, and may provide evidence of disease-related changes. (Pang et al., 2002).

**Fig. 1:**
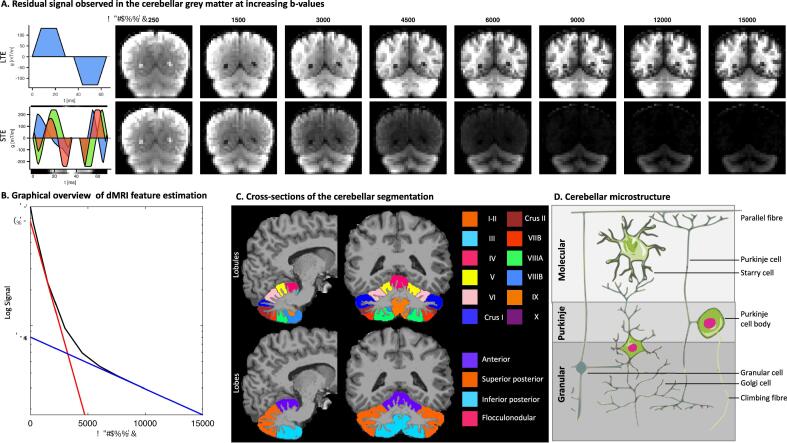
**A**. Linear b-tensor diffusion encoding (LTE, top) with diffusion sensitisation along a single axis, and spherical b-tensor diffusion encoding (STE, bottom) with diffusion sensitisation in all directions. Timings for the first waveform, temporal gap (180° pulse), and second waveform were [28.6, 6.9, 28.6] ms for the linear encoding and [35.5, 6.9, 25.6] ms for STE, respectively. The figure show averages across DWIs per b-value, with the intensity min–max normalised per b-value according to the LTE image. **B.** Graphical overview of the estimation of dMRI features from the STE signal as a function of b-value (black line), here simulated as a tri-exponential decay with $f=\left[0.2,0.72,0.08\right]$ and $D=\left[3,1,0.1\right]\mu m^{2}/ms$ where the first and last compartment mimic free water and a spherical restricted compartment, respectively. The blue line is estimated by fitting a mono-exponential function to b ​≥ 10,000 s/mm^2^ and has slope $D_{s}$ and y-intercept $S_{0,s}$. The red line is estimated by fitting a bi-exponential function $S=S_{0}\left(f_{t}\exp\left(-b\cdot MD\right)+\left(1-f_{t}\right)\exp\left(-b\cdot 3\right)\right)$ to b ​≤ 1500 s/mm^2^ and has slope MD and y-intercept $f_{t}S_{0}$. At low b-values, the deviation from mono-exponential behaviour is assumed to be arising from the free water compartment with a diffusivity of $3\mu m^{2}/ms$. **C.** Cerebellar segmentation in lobules (top) and lobes (bottom). **D.** Schematic cross-sectional representation of cerebellar grey matter microstructure including the granule cell layer (yellow), Purkinje cell layer (grey) and the molecular layer (blue). (For interpretation of the references to colour in this figure legend, the reader is referred to the web version of this article.)

In recognition of these limitations, more advanced imaging methods have been proposed that aim to enhance the specificity of dMRI. (Alexander et al., 2019) One recent example has been a focus on the proportional signal contribution from small spherical spaces, and its potential role as a proxy measurement of cellular density. (Palombo et al., 2020, Tax et al., 2020) Assessment of this signal contribution can be improved by the use of so-called ‘spherical tensor encoding’ (STE) as it is sensitive to diffusion in all directions in a single measurement. (Westin et al., 2016) This provides complementary information to the conventional ‘linear tensor encoding’ (LTE), widely used in DTI studies, for example, which provides sensitivity to diffusion only along a single axis (Fig. 1A). (Mori and Van Zijl, 1995, Eriksson et al., 2013) At ultra-high diffusion-weightings, STE suppresses signal from water pools in elongated structures in which diffusion takes place along at least one axis, such as axons, and is particularly suited to isolating signals from compartments with very low diffusivity in all directions – e.g. small spherical spaces or a ‘dot’ compartment with zero apparent diffusivity. Previous work using this technique has shown a consistent residual signal in the cerebellar GM of healthy controls at very high diffusion-weightings (Fig. 1A), indicating a potential role in detecting cerebellar GM microstructural changes, (Tax et al., 2020, Lundell et al., 2019, Vis et al., 2021) and of potential utility in detecting changes of cerebellar GM microstructure in movement disorders where a role for the cerebellum has been implicated. (Burciu et al., 2017, Adanyeguh et al., 2018, Selvadurai et al., 2020, Maiti et al., 2020) The residual signal itself is derived from isotropically restricted water compartment, previously shown to be present in cerebellar GM, which at high b-values results in a residual yet slowly decaying signal above the noise floor.

This study applies these approaches *in vivo* to image cerebellar GM microstructure in human participants diagnosed with an underlying neurological disorder, providing proof-of-concept of enhanced specificity and sensitivity to cerebellar GM microstructural changes in movement disorders. For this proof-of-concept, we applied this technique to three distinct movement disorder types – adult-onset, idiopathic, focal cervical dystonia (AOIFCD), referred to as dystonia from this point forward, Parkinson’s disease (PD) and Spinocerebellar Ataxia type 6 (SCA6) - spanning neurodegenerative and neurodevelopmental disorders, with varying degrees of evidence for involvement of cerebellar pathology. Previous work has demonstrated evidence of reduced GM Purkinje cell density and impaired dendritic trees, as well as a reduced number of dendritic spines in both post-mortem and animal model work of these disorders. (Prudente et al., 2013, LeDoux, 2011, Wang et al., 2010, Jayabal et al., 2017) Where possible, we have sought to relate distinct isotropic diffusion patterns to known regions of histopathological change, and to investigate the extent to which this technique may be of future prognostic and treatment monitoring value.

## 2 Methods

### 2.1 Participant recruitment and data collection

Participants were recruited via the Welsh Movement Disorders Research Network (REC ref: 14/WA/0017, IRAS ID: 146495). Informed consent was obtained, with ethical approval provided by the Cardiff University School of Medicine Research Ethics committee (ref: 18/30, ‘Investigating the use of Magnetic Resonance Imaging (MRI) in understanding disease mechanisms in individuals with movement disorders’). Standardised questionnaires were used to collect baseline clinical data and all genetic mutations were confirmed in NHS genetics facilities by next generation or direct sequencing techniques. Two unaffected control groups were recruited, the first (Group 1), was imaged on two separate occasions to determine the reproducibility of the microstructural imaging findings, and the second (Group 2) age-matched to the patient cohorts. Details relating to a subset of the non-age matched healthy control participants have been reported previously. (Tax et al., 2020) All participants underwent MRI brain scanning on a 3 T ultra-strong gradient (300 mT/m) MRI system (Siemens Healthcare, Erlangen, Germany).

### 2.2 Data acquisition and processing

#### 2.2.1 Acquisition

The acquisition protocol included a structural Magnetization Prepared RApid Gradient Echo (MPRAGE) with voxel size 1​×1​×1 mm, and dMRI sequences. The dMRI data were acquired using a prototype spin-echo sequence (Szczepankiewicz et al., 2019) with an echo-planar imaging (EPI) readout that enables user-defined gradient waveforms to be used for diffusion encoding (Fig. 1A). (Tax et al., 2020, Szczepankiewicz et al., 2019) STE was performed with b ​= ​[250, 1500, 3000, 4500, 6000, 7500, 9000, 10500, 12​000, 13500, 15000] s/mm^2^, and repeated Adanyeguh et al., 2018, Alexander et al., 2019, Bammer et al., 2003, Glasser et al., 2013, Jayabal et al., 2017, Jenkinson and Smith, 2001, Koay et al., 2009, Lundell et al., 2019, Prudente et al., 2013, Vos et al., 2017, Westin et al., 2016 times, respectively. The acquisition order of b-values and repetitions was randomized to reduce the impact of system drift. (Vos et al., 2017) For conventional diffusion encoding (pulsed-gradient spin echo, or LTE), (Adanyeguh et al., 2018, Szczepankiewicz et al., 2021) the b-tensor principal eigenvectors were distributed over the unit sphere for each b-shell, where the number of directions aand b-values equalled the number of repeats and b-values in STE, respectively. b ​= ​0 s/mm^2^ (b0) images were acquired every 15th image for monitoring and correction of subject motion. Additional b = 0 s/mm^2^ images with reversed phase-encoding were acquired to correct for susceptibility distortions. No in-plane acceleration was used, and imaging parameters were: voxel size ​= ​4​×4×​4 mm^3^ to achieve high signal-to-noise ratio, matrix ​= ​64 × 64, 34 slices, TE = 88 ms, TR ​= ​4300 ms, partial-Fourier ​= ​6/8, bandwidth ​= ​1594 ​Hz/pixel. Total acquisition time was 36 min.

#### 2.2.2 Diffusion MRI data processing

The dMRI data were checked for signal intensity outliers (Sairanen et al., 2018) and pre-processed as follows. Rician noise bias correction was performed to reduce effects of the noise floor leading to an artificial signal plateau in the signal decay curve. (Koay et al., 2009) The data were corrected for signal drift, (Vos et al., 2017) Gibbs ringing, (Kellner et al., 2016) image-misalignment due to subject motion and eddy currents by an initial rigid registration according to the interleaved b0 images, and a subsequent affine registration of each diffusion weighted image (DWI) to the mean per b-value. All data were corrected for geometrical distortions due to susceptibility effects (Andersson et al., 2003) and gradient nonlinearities. (Glasser et al., 2013, Jones et al., 2018, Setsompop et al., 2013) Spatially varying effective b-values to account for gradient nonlinearities were also computed. (Bammer et al., 2003).

Various dMRI features were estimated from the data. Under the assumption of multiple non-exchanging Gaussian diffusion compartments (each represented by a diffusion tensor), the STE diffusion signal as a function of b-value can be written in terms of a sum of the signal fractions and mean apparent diffusivities of each compartment. At high b-values (b ​≥ 10,000 s/mm^2^), the remaining signal arises from small spherical compartments with extremely low diffusivities, or ‘dot-like’ compartments, (Tax et al., 2020) and this signal can be used to estimate the signal and apparent diffusivity associated with such a compartment (denoted as $S_{0,s}$ and $D_{s}$, respectively, Fig. 1B). At low b-values (b ​≤ 1500 s/mm^2^), the STE signal can be used to estimate the mean apparent diffusivity (MD) as opposed to deriving it from DT-MRI. (Mori and Van Zijl, 1995) MD reflects a weighted average of apparent diffusivities across all contributing compartments (including the spherical compartment). In addition, to reduce CSF-contributions, which can significantly bias MD estimates in case of e.g., atrophy, a free-waterelimination strategy was adopted. (Pasternak et al., 2009) Briefly, a bi-exponential decay was fitted to the low b-value data, where one compartment represents CSF (with a fixed diffusivity of $3\;\mu m^{2}/ms$) and the other represents tissue (for which the diffusivity is not fixed, but estimated). From this, the free-water-eliminated tissue MD, tissue fraction $f_{t}$ and $b=0\;s/mm^{2}$ signal $S_{0}$ were estimated. The free-water-eliminated tissue signal fraction of the spherical compartment was then computed as $f_{s}=S_{0,s}/\left(f_{t}S_{0}\right)$. All fits were performed with a nonlinear-least squares trust-region-reflective algorithm, where signals with a modified Z-score larger than 3.5 were excluded as outliers. (Sairanen et al., 2018).

#### 2.2.3 Cerebellar segmentation

The MPRAGE image was used to segment the cerebellum (Romero et al., 2017) and was affinely registered to the corrected b0-image to obtain segmentations of the dMRI images. (Jenkinson and Smith, 2001) Segments were grouped into lobes: anterior (A); superior posterior (SP); inferior posterior (IP); Flocculonodular (F) (Fig. 1C). Only GM voxels with $f_{t}>0.3$ and $f_{s}$- and $D_{s}$-values within the 99th percentile were included in further analysis.

### 2.3 Statistical analysis

Median values for $f_{s}$, $D_{s}$, and MD were computed for each cerebellar lobule (I-IX, Fig. 1C top). To calculate the median values per lobe, voxels for all lobules within the lobe were pooled from which median values were calculated per lobe (Fig. 1C bottom). Mann-Whitney tests were used for comparison of the two control groups (control group 1 vs. control group 2) and comparison of basic demographic characteristics between all groups. Intraclass correlation coefficient used to determine the reproducibility of the microstructural imaging findings, calculated using data collected from control group 2 (non-age-matched unaffected participants, n = 5) imaged on two separate occasions. Movement disorder groups were compared to the age-matched unaffected control group 2 using a multiple linear regression model, controlling for total cerebellar volume, for the overall cerebellar analyses, and one-way ANOVA test with post-hoc Tukey’s test for comparison of the individual cerebellar lobes (anterior, superior posterior and inferior posterior). Bonferroni correction for multiple metrics was applied to all between group analyses.

## 3 Results

### 3.1 Cohort demographic characteristics

Fifteen participants diagnosed with a movement disorder (10F:5M, 56.8 ± 10.42 years), 10 non-age-matched, unaffected control participants (6F:4M, 27.4 ± 5.64 years) (Control Group 1) and five age-matched unaffected controls (3F:2M, 51.4 ± 6.27 years) (Control Group 2) were recruited. The movement disorder cohort included those diagnosed with idiopathic Parkinson’s disease (PD) (n = 5), Dystonia (n = 5) and SCA6 (n = 5), with no significant differences in age between Control Group 2 and each of the movement disorder cohorts (PD: p = 0.83, Dystonia: p = 0.09, SCA6: p = 0.21). (Table 1, Table 2).

**Table 1:** Comparison of morphometric and grey matter microstructural properties between control groups of differing age.

	**Control Group 1**	**Control Group 2**	**p-value**
A. **Demographic Characteristics**			
n	10	5	
Sex (M: F)	4:6	2:3	0.58
Age at examination (median (range))	24.5 (20–39)	51 (43–58)	0.003*
B. **Morphometry (Median (Median Absolute Deviation))**			
Cerebellar volume (total) cm^3^	132.81 (12.05)	115.94 (21.02)	0.36
Cerebellar Grey Matter ROI volume (cm^3^)	99.14 (9.34)	85.01 (14.29)	0.17
Cerebellar Grey Matter ROI thickness (mm)	(0.10)	4.59 (0.04)	0.22
C. **Grey Matter Microstructure (Median (Median Absolute Deviation))**			
***Cerebellum (Overall)***			
Mean Diffusivity (MD) μ*m*√^2^/*ms*	0.61 (0.04)	0.60 (0.03)	0.36
Sphere Signal Fraction ($f_{s}$)	0.11 (0.02)	0.11 (0.01)	0.17
Sphere Diffusivity ($D_{s})$ μ*m*√^2^/*ms*	0.13 (0.02)	0.12 (0.02)	0.22
***Anterior Lobe***			
Mean Diffusivity (MD) μ*m*√^2^/*ms*	0.65 (0.03)	0.62 (0.04)	0.01
Sphere Signal Fraction ($f_{s}$)	0.11 (0.02)	0.10 (0.01)	0.88
Sphere Diffusivity ($D_{s})$ μ*m*√^2^/*ms*	0.14 (0.03)	0.13 (0.01)	0.32
***Superior Posterior Lobe***			
Mean Diffusivity (MD) μ*m*√^2^/*ms*	0.61 (0.03)	0.60 (0.03)	0.72
Sphere Signal Fraction ($f_{s}$)	0.10 (0.01)	0.10 (0.01)	0.14
Sphere Diffusivity ($D_{s})$ μ*m*√^2^/*ms*	0.12 (0.09)	0.12 (0.01)	<0.001*
***Inferior Posterior Lobe***			
Mean Diffusivity (MD) μ*m*√^2^/*ms*	0.59 (0.053)	0.59 (0.03)	0.49
Sphere Signal Fraction ($f_{s}$)	0.13 (0.02)	0.11 (0.03)	0.005
Sphere Diffusivity ($D_{s})$ μ*m*√^2^/*ms*	0.14 (0.01)	0.13 (0.02)	0.07

ROI: Region of Interest, Bonferroni corrected p-value to correct for multiple comparisons < 0.003.

**Table 2:** Demographic data, Cerebellar Grey Matter Morphometric and Microstructural Analysis.

	**Control Group 2**	**Parkinson’s disease**	**Dystonia**	**SCA6**
**A. Demographic Characteristics**				
n	5	5	5	5
Sex (M: F)	2:3	3:2	2:3	0:5
Age at examination (median (range)) [p-value: comparison to control]	51 (43–58)	54 (36–57) 0.83	59 (53–60) 0.09	65 (46–82) 0.21
**B. Morphometry (**Median (Median Absolute Deviation))				
Cerebellar volume (total) cm^3^	115.94 (21.02)	127.64 (19.91) [p = 0.75]	101.67 (6.14) [p = 0.98]	89.60 (12.90) [p = 0.16]
Cerebellar Grey Matter ROI volume (cm^3^)	85.01 (14.29)	95.02 (17.98) [p = 0.94]	80.29 (5.55) [p = 0.95]	64.93 (11.39) [p = 0.13]
Cerebellar Grey Matter ROI thickness (mm)	4.59 (0.04)	4.65 (0.05) [p = 0.54]	4.55 (0.06) [p = 0.99]	4.12 (0.25) **[p = 0.001]**
**C. Grey Matter Microstructure (**Median (Median Absolute Deviation), [p-value])				
***Cerebellum (Overall)***				
Mean Diffusivity (MD) μm^2^/ms	0.60 (0.03)	0.58 (0.03)	0.55 (0.06)	0.54 (0.07)
		[p = 0.01]	**[p = 1.04e-08]**	**[p = 3.29e-07]**
Sphere Signal Fraction ($f_{s}$)	0.11 (0.01)	0.11 (0.02)	0.13 (0.03)	0.14 (0.03)
		[p = 0.01]	**[p = 3.94e-08]**	**[1.27e-05]**
Sphere Diffusivity ($D_{s})$ μm^2^/ms	0.12 (0.02)	0.13 (0.02)	0.14 (0.013)	0.13 (0.02)
		[p = 0.33]	[p = 0.02]	[p = 0.05]
***Anterior Lobe***				
Mean Diffusivity (MD) μm^2^/ms	0.62 (0.04)	0.62 (0.06) [p = 0.84]	0.55 (0.09) [p = 0.04]	0.60 (0.07) [p = 0.27]
Sphere Signal Fraction ($f_{s}$)	0.10 (0.01)	0.10 (0.03) [p = 0.50]	0.13 (0.03) [p = 0.04]	0.11 (0.03) [p = 0.66]
Sphere Diffusivity ($D_{s})$ μm^2^/ms	0.13 (0.01)	0.12 (0.02) [p = 0.55]	0.15 (0.02) [p = 0.02]	0.11 (0.03) [p = 0.27]
***Superior Posterior Lobe***				
Mean Diffusivity (MD) μm^2^/ms	0.60 (0.03)	0.59 (0.03) [p = 0.05]	0.57 (0.05) **[p = 4.42e-05]**	0.54 (0.06) **[p = 6.70e-05]**
Sphere Signal Fraction ($f_{s}$)	0.10 (0.01)	0.10 (0.02) [p = 0.05]	0.12 (0.03) **[p = 6.81e-05]**	0.13 (0.02) **[p = 8.47e-04]**
Sphere Diffusivity ($D_{s})$ μm^2^/ms	0.12 (0.01)	0.13 (0.10) [p = 0.17]	0.13 (0.10) [p = 0.53]	0.12(0.01) [p = 0.03]
***Inferior Posterior Lobe***				
Mean Diffusivity (MD) μm^2^/ms	0.59 (0.03)	0.57 (0.03) [p = 0.03]	0.52 (0.09) **[p = 2.06e-04]**	0.51 (0.06) [p = 0.004]
Sphere Signal Fraction ($f_{s}$)	0.11 (0.03)	0.11 (0.02) [p = 0.05]	0.15 (0.04) **[p = 4.27e-04]**	0.16 (0.03) [p = 0.001]
Sphere Diffusivity ($D_{s})$ μm^2^/ms	0.13 (0.02)	0.14 (0.02) [p = 0.65]	0.14 (0.01) [p = 0.30]	0.13 (0.01) [p = 0.76]

**A:** F: Female, M: Male **B:** Morphometric measurements for each subgroup within the study cohort, ROI: Region of Interest, **C:** Median and Median Absolute Values for each imaging measure for each of the cohort subgroups. One-way ANOVA with post-hoc Tukey’s test used for comparison of movement disorder groups to unaffected control cohort with subsequent Bonferroni correction for multiple metric comparisons (p < 0.003).

### 3.2 Reproducibility of microstructural measures

**Fig. 2:**
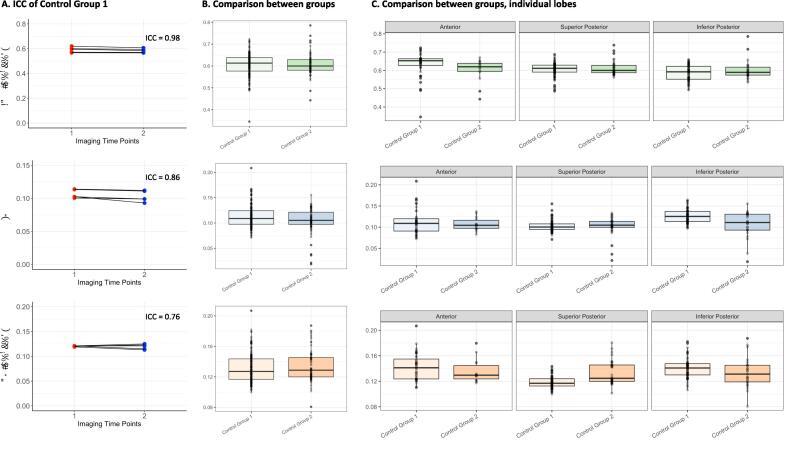
**A.** Intraclass correlation coefficient (ICC) for different dMRI measures (rows) in Control Group 1. **B.** Comparison of estimated dMRI measures between Group 1 and 2 across lobes. **C.** Comparison of estimated dMRI measures between Group 1 and 2 for different lobes.

Five participants of the non-age-matched control cohort (Control Group 1) underwent repeat imaging at a subsequent time point to ensure the consistency of these novel measurements. High levels of reproducibility were demonstrated across the cerebellum for all measures ($MD,\;f_{s}$ and $D_{s}$), with intraclass correlation coefficients (ICC) of 0.98, 0.86 and 0.76, respectively (Fig. 2A). The flocculonodular lobe was removed from ongoing analysis due to the low number of voxels identified in comparison to both anterior and posterior lobes (<5 for all control participants).

### 3.3 Comparison of microstructural measures between different control groups

Control Groups 1 and 2 differed in median age at examination (24.5 and 51 years respectively) (Table 1). No significant differences were observed in their overall cerebellar morphometric properties, calculated from T1-weighted images, including cerebellar volume (p = 0.36), cerebellar grey matter volume (p = 0.17) and cerebellar cortical thickness (p = 0.22). Analysis of the cerebellar GM microstructural measurements for the whole cerebellum also found no significant differences between the two control groups for *M* D (p = 0.36), $f_{s}$ (p = 0.17) and $D_{s}$ (p = 0.22) (Fig. 2B). However, when assessing the individual cerebellar lobes, a significantly higher $D_{s}$ in the superior posterior lobe (p < 0.001) was observed in the older control group (Group 2), compared to that of the younger unaffected control group (Control Group 1), but no significant differences for any of the remaining individual lobe measures (Table 1, Fig. 2C).

### 3.4 Comparison of movement disorders cohorts and age-matched controls

#### 3.4.1 Cerebellar morphometric Characteristics

Comparison of total cerebellar volume, cerebellar GM volume and thickness found no significant differences between the unaffected control group and each of the movement disorders examined (PD, Dystonia and SCA6) following Bonferroni correction for multiple metric comparisons with the exception of lower cerebellar GM thickness in the SCA6 cohort compared to controls (p = 0.001) (Table 2B). It should be noted however, that although multiple measures were applied to minimise the potential for partial volume mixture with CSF and WM, these may have contributed to these estimated values.

#### 3.4.2 Cerebellar grey matter micrpstructural properties

**Fig. 3:**
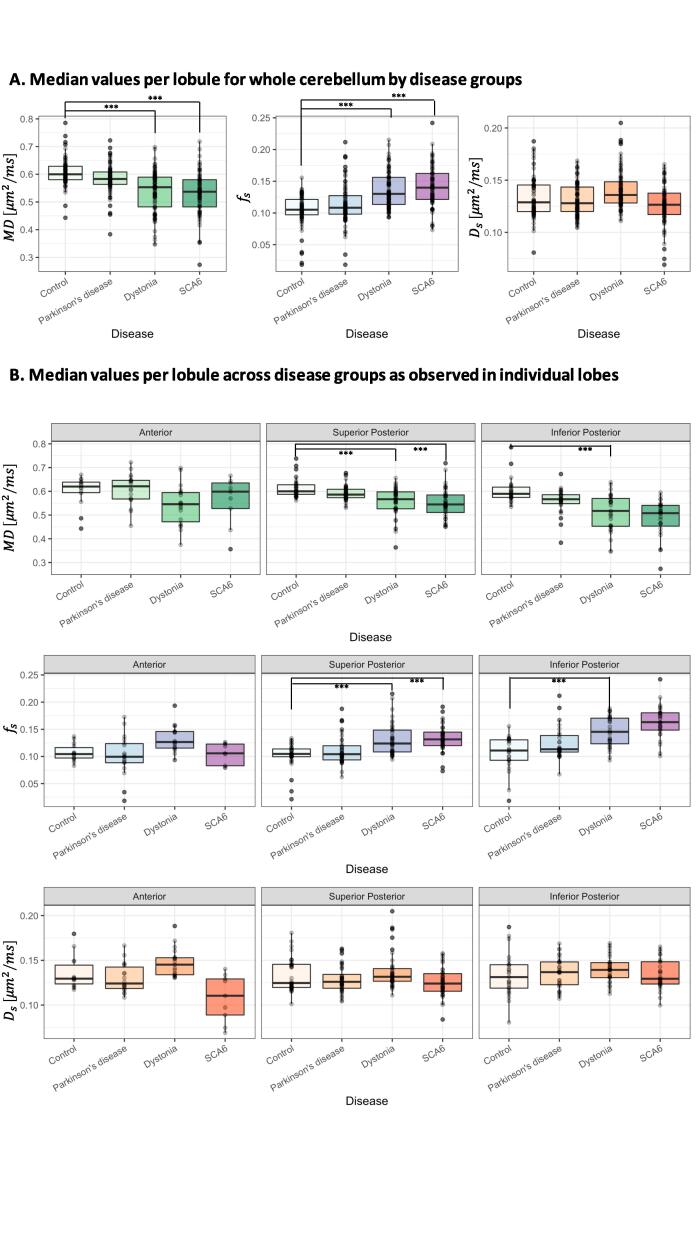
**A**. Boxplots of the median values of mean diffusivity (MD), signal fraction ($f_{s})$ and diffusivity ($D_{s}$) from each cerebellar region (I-X) (circles) across the whole cerebellum, excluding the flocculonodular lobe. **B**. Boxplots of the median values for each voxel within each region (I-X) (circles) analysed by cerebellar lobe; anterior, superior posterior and inferior posterior. *** denotes statistically significant comparisons, beyond that of the Bonferroni correction for multiple metric comparisons (p < 0.003).

Analysis of the median voxel values across anterior and posterior cerebellar lobes identified significantly lower MD in SCA6 (p = 3.29e-07) and Dystonia (p = 1.04e-08) cohorts (Table 2C, Fig. 3A), while significantly higher $f_{s}$ values were observed in the same two movement disorder groups, Dystonia and SCA6 (p = 3.94e-08 and p = 1.27e-05 respectively). No significant differences were observed with $D_{s}$ measurements between the individual movement disorder groups and the control cohort.

#### 3.4.3 Analysis of individual lobes

Analysis of individual lobes identified significantly different values only in the Dystonia and SCA6 cohorts compared to controls, with these differences limited to the superior and inferior posterior lobes in the dystonia cohort, superior posterior lobes alone in the SCA6 group, and no significant changes across any of the disorders in the anterior lobes (Table 2C). Superior posterior cerebellar lobe MD values were found to be significantly lower in dystonia (p = 4.42e-05) and SCA6 (p = 6.70e-05) groups compared to controls while the $f_{s}$ was significantly higher (dystonia, p = 6.81e-05; SCA6, p = 8.47e-04). A similar pattern was observed for dystonia in the inferior posterior lobe with significantly lower MD values (p = 2.06e-04) and higher $f_{s}$ values (p = 4.27e-04) No significant differences were observed in the $D_{s}$ values across any of the individual lobes (Table 2C, Fig. 3B).

## 4 Discussion

Primarily due to a lack of appropriate MRI methods Cerebellar GM microstructure has been under-investigated *in vivo* to date. This is the first application of STE dMRI at ultra-high b-values with a potential imaging proxy for cerebellar GM cell body density in a movement disorder patient cohort. A range of degenerative and non-degenerative movement disorders were examined, including those associated with predominant cerebellar dysfunction (e.g. SCA6), those where the cerebellum is recognised to play a contributing role (e.g. Dystonia), and those known to demonstrate more diffuse degenerative changes (e.g., Parkinson’s disease). (Burciu et al., 2017) In so doing, we have demonstrated consistent variation in cerebellar grey matter microstructure in degenerative (SCA6) and non-degenerative (Dystonia) movement disorders, relative to control populations. This study provides a promising initial step, from which work can be extended to larger cohorts and more detailed analysis of the cellular variation contributing to the observed signal change.

Conventional measures, such as MD, from DTI reflect the overall presence of microstructural barriers to diffusion and could pre-date morphometric volume changes, such as previously reported MD changes identified prior to cortical thinning in Alzheimer’s disease, (Spotorno et al., 20232023) potentially providing a useful measure of early GM cellular changes. Consistent with previous studies in cerebellar WM, (Sondergaard et al., 2021) lower GM MD has been observed in those with dystonia, with the changes identified in this study suggested by the authors to be mainly in axonal properties. By contrast the associated increase in $f_{s}$ values observed here suggest additional changes in small spherical (and thus non-axonal) compartments, extending the potential source of these signal changes to other microstructural components. Previous studies have not reported changes in the MD of cerebellar WM or GM in Parkinson’s Disease, but have identified lower FA in cerebellar GM compared to controls. (Zhang et al., 2011) Moreover, a recent review and *meta*-analysis of DTI findings in PD identified FA and MD values to be differentiating between those with PD and controls when examining the substantia nigra, corpus callosum, cingulate and temporal cortices but not for cerebellar measures. (Atkinson-Clement et al., 2017) In apparent contrast with our results, increased MD values have been reported in the cerebellar WM of SCA6 cases, (Park et al., 2020) however, it should be noted that this previous work does not report correction of MD for free water as done in this study, potentially contributing to conflicting results.

Recent *in vivo* imaging studies have provided further support for a central role for the cerebellum in dystonia pathogenesis, and more specifically in adult-onset dystonic tremor, where fMRI analysis demonstrated increased activity bilaterally in the cerebellum and cerebello-thalamic pathway. (Nieuwhof et al., 2022) In this study, average MD values were lower in those with Dystonia compared to controls, suggesting increased structural density. The biological correlates remain unknown; however, human post-mortem and murine models point towards a similar cellular region with predominant involvement of the Purkinje Cell layer, although granule cells are more numerous and may too contribute, potentially to a larger degree, to the observed signal (Fig. 1D). Animal models of dystonia have identified increased numbers of synaptic boutons on the dendritic shafts and soma of Purkinje cells in the molecular layer of the cerebellar cortex, with immunostaining suggesting that these boutons were derived from GABAergic interneurons. (Inoue et al., 1993) By contrast, the limited number of human post-mortem studies in those with Dystonia have demonstrated lower overall Purkinje Cell density, while the murine “wriggle mouse sagami” model has demonstrated impaired development of Purkinje Cell dendritic trees with fewer synaptic connections from parallel fibres to the Purkinje cells. (Prudente et al., 2013, LeDoux, 2011) Transgenic models of DYT1 (*TorsinA*) dystonia have shown similar findings in Purkinje Cell morphology, with shortened primary dendrites and a decreased number of spines on distal dendrites. (Zhang et al., 2011) Finally, targeted loss of Purkinje cells in the *tottering^PC-loss^* mouse results in a dystonic phenotype, with dystonia severity correlated with the linear density of Purkinje Cells throughout the cerebellum. (Raike et al., 2013) Notably, however, size estimates of the tissue represented by the dot signal in our previous work placed this in the range of granule cells within the cerebellum. While only a limited number of transgenic animal models have focused on these specific cell types, those harbouring dystonia gene, *PRRT2* mutations (DYT10), found that those with mutations limited to the granule cells (GCs) recapitulated the behavioural phenotypes seen in *Prrt2*-null mice, with optogenetic stimulation of granule cells resulting in transient elevation followed by suppression of Purkinje cell firing. (Tan et al., 2018).

A significant pattern of higher $f_{s}$ was also observed in the SCA6 cohort. SCA6 is an autosomal dominant, late-onset cerebellar ataxia, caused by trinucleotide (CAG) expansion in the alpah1A voltage-gated calcium channel gene (*CACNA1A*), the wild-type protein of which is highly expressed in both cerebellar Purkinje and granule cells. In addition, human post-mortem studies in those with SCA6 mutations have demonstrated fewer, thicker branches, and a reduced number of Purkinje Cell dendritic spines, with similar microstructural changes in transgenic animal models, once again suggesting that the signal observed in this study emanates at a cellular level from the Purkinje or granule cells of the cerebellum (Wang et al., 2010, Jayabal et al., 2017) However, the clear difficulty in this explanation is that while histopathological examination of dystonia models suggests an increase in cellular density, which in some cases is correlated with increasing motor symptom severity, here, the signal changes associated with SCA6 would be more consistent with reduced cellular density. This raises the possibility that the signal changes observed here, in the SCA6 cohort, may reflect small spherical compartments generated by cellular degeneration but not occupied by a specific cellular subtype.

Future evaluation using this approach in a larger cohort may further improve interpretation on a disease-specific level, particularly changes to the dot signal fraction in other cerebellar-predominant degenerative disorders and inference as to the underlying histological change that this may represent. Furthermore, examination of changes to the diffusion of metabolites (which tend to be contained entirely in the intra-cellular space) could help reveal whether the isotropically-restricted diffusion-weighted signal predominantly arises from the intra- or extra-cellular space, further refining the tissue type from which it is derived. Furthermore, the present analysis explicitly assumed the signal to originate from non-exchanging compartments. However, recent work suggests relatively high exchange rates in gray matter in general and in cerebellar gray matter in particular. (Olesen et al., 2022) As a consequence, we expect the values of $f_{s}$ to be lower than expected from histology and protocol dependent, as different gradient waveforms have different sensitivity to exchange, as well as there being potential bias due to microscopic GM kurtosis. (Chakwizira et al., 2023, Novello et al., 2022) The former point also indicates that a change in $f_{s}$ can be interpreted either as a change in its corresponding volume fraction or a change in the membrane permeability of the related cell population. The coarse spatial resolution was adopted to maintain acceptable signal-to-noise ratio (SNR) in the diffusion-weighted data, and despite the highly curved structure of the cerebellar GM, partial volume mixture with CSF and WM was minimised by the use of cerebellar segmentation, free-water elimination, and high b-value STE. However, methodological choices (e.g., the b-values used in the free-water elimination step) and potential inaccuracies (e.g., subtle misalignment with the T1-weighted scan or segmentation inaccuracy) could still affect the results, and future work should strive to investigate this into greater detail. A potential solution to achieving high spatial resolution while preserving the SNR is to adopt alternative image acquisition strategies. Vis et al. recently demonstrated that a similar image contrast can be achieved at 1.6 mm isotropic resolution by using STE at b = 4000 s/mm^2^ paired with a super resolution image reconstruction. (Vis et al., 2021).

## 5 Conclusion

In summary, this study suggests a promising, non-invasive, *in vivo* measure of cerebellar GM microstructural differences across degenerative and non-degenerative movement disorders. We have also demonstrated the potential for enhancing sensitivity and specificity to small spherical spaces, directly from the high b-value diffusion-weighted STE signal, rather than the more time consuming, costly, and potentially biased modelling of all compartments. Future work will involve further elucidating the microstructural origins of the residual signal, with the which could originate from within small spaces that may be intra-cellular (e.g. dendritic spines or dendritic arbor) or extra-cellular (e.g. between densely packed granule cells), or both.

## 6 Data statement

The raw neuroimaging data that support the findings of this study are available upon request from the corresponding author and following agreement with the host institution (Division of Psychological Medicine and Clinical Neurosciences, Cardiff University).

## Declaration of Competing Interest

The authors declare that they have no known competing financial interests or personal relationships that could have appeared to influence the work reported in this paper.

## Data Availability

Data will be made available on request.
